# Occurrence of ochratoxin A in breast milk and urine samples of nursing mothers in Bangladesh

**DOI:** 10.1007/s12550-023-00510-5

**Published:** 2023-12-01

**Authors:** Aporajita Das Trisha, Jaasia Momtahena Hafsa, Akibul Hasan, Ahsan Habib, Humaira Rashid Tuba, Gisela H. Degen, Nurshad Ali

**Affiliations:** 1 Department of Biochemistry and Molecular Biology, Shahjalal University of Science and Technology, Sylhet, 3114 Bangladesh; 2 Leibniz-Research Centre for Working Environment and Human Factors (IfADo) at the TU Dortmund, Ardeystr. 67, D-44139 Dortmund, Germany

**Keywords:** Bangladesh, Biomarker, Breast milk, Contaminant, Exposure, Infants, Mycotoxin, Ochratoxin A

## Abstract

The mycotoxin ochratoxin A (OTA) is a potent nephrotoxin with carcinogenic properties and, thus, of concern as a food contaminant. Since food contaminant data are scarce in Bangladesh, we applied human biomonitoring to gain more insights into OTA exposure in the country’s population. OTA concentrations in human milk and urine samples of nursing mothers were determined with the aim to assess also exposure to this mycotoxin in breastfed infants. Breastfeeding mothers (n = 74) from three districts of Bangladesh (Sylhet, Cumilla, and Mymensingh region) participated in this study. They provided demographic data, along with breast milk and urine samples. OTA levels were measured by a competitive enzyme-linked immunosorbent assay (ELISA) with a detection limit of 60 ng/L for milk and 30 ng/L for urine.

OTA was detected in 62.2% of all breast milk samples (mean 74.8 ± 49.0 ng/L, range < LOD–243.3 ng/L) and in 51.4% of all urine samples (mean 44.3 ± 63.5 ng/L, range < LOD–519.3 ng/L). The differences observed between regions for mean breast milk or for urinary OTA levels were relatively small. No significant correlation was observed between OTA levels in breast milk and food consumption patterns among nursing mothers. Regarding infant exposure, the estimated average daily intake of OTA for all was 15.0 ng/kg bw/day (range 4.5–45 ng/kg bw/day). In 34.5% of these infants, their estimated daily OTA intake exceeded a preliminary TDI value set by EFSA (17 ng/kg bw/day). The mean OTA intake was slightly higher (16.2 ± 7.8 ng/kg bw/day) in 1–2 months babies than in older infants (< 2 to 12 months), although the difference was not significant. Presence of OTA in most milk and urine samples of nursing mothers documents their widespread dietary mycotoxin exposure. Although based on a relatively small number of participants, the present analysis indicates non-negligible exposure of some nursed infants in Bangladesh. Therefore, further biomonitoring studies and investigations on major sources of OTA in food commodities are encouraged.

## Introduction

Ochratoxin A (OTA) is a secondary fungal metabolite produced by some species of the genera *Aspergillus* and *Penicillium* in many parts of the world (Frisvad et al. 2006). OTA has raised significant concerns for human as well as animal health due to its widespread occurrence in many foodstuffs (Ostry et al. 2013). It can cause nephrotoxicity in various species, and has been identified as potent renal carcinogen in rodents (EFSA 2006; Mally and Dekant 2009). Contamination of food commodities with mycotoxins including OTA cannot be avoided completely. Yet, scientific advisory committees have derived tolerable intake levels (between 3 and 17 ng/kg bw/day) for OTA in humans (EFSA 2006; FAO/WHO 2007; Kuiper-Goodman et al. 2010). Human exposure to mycotoxins is assessed by *i)* analysis of contaminant levels in relevant foodstuffs combined with food consumption data, and *ii)* analysis of mycotoxin biomarker levels in human body fluids such as blood, urine and breast milk (Duarte et al. 2011; Ali et al. 2016a). Among these approaches, the second one is most effective as it reflects an exposure from all sources for individuals of a given cohort (Degen 2011; Duarte et al. 2011; Fromme et al. 2016; Turner et al. 2012).

Breast milk is the ideal form of nutrition for infants and important for their growth and health (Degen et al. 2013). But, toxic compounds, including mycotoxins ingested by nursing women can get into milk (Cherkani-Hassani et al. 2016; Warth et al. 2016; Degen et al. 2017). Although levels of OTA in breast milk are lower than those found in the maternal blood (Muñoz et al. 2014), its presence in human milk represents a health hazard for newborns and infants. Of note, infants are considered as vulnerable group due to immature detoxication and excretion mechanisms and a higher food intake per body weight than adults (Degen et al. 2013; EFSA Contam Panel et al. 2020). In addition to human milk, urine is also an appropriate matrix for investigating OTA exposure by non-invasive sampling procedures. OTA analysis in urines better reflects day-to-day variations in exposure of adults and infants, though only a small fraction of ingested OTA is excreted in urine (Ali et al. 2017a; Degen 2016; Gilbert et al. 2001; Muñoz et al. 2014).

Occurrence of OTA in human breast milk has been documented in several studies, with considerable differences in toxin levels between cohorts from different countries in Africa, Asia, Europe and South America (Cherkani-Hassani et al. 2016). Other studies indicated the frequent presence of OTA in urine samples of young children, pregnant women and adults in Bangladesh (Ali et al. 2016a, b; Ali and Degen 2020; Kyei et al. 2022). However, OTA occurrence in human breast milk in Bangladesh has not been investigated so far, although OTA biomarker analysis in nursing mothers is of particular interest. Therefore, the aim of our study was to measure the OTA concentrations in both breast milk and maternal urine samples and assess also infant exposure to this mycotoxin in Bangladesh.

## Materials and methods

### Study areas and study participants

This cross-sectional study was conducted between March 2021 and November 2021, the period of recruiting volunteers and collection of samples. All related analyses were performed at the Department of Biochemistry and Molecular Biology of Shahjalal University of Science and Technology, Sylhet, Bangladesh. A total of 74 lactating mothers from three districts region (Sylhet, Cumilla, Mymensingh) of Bangladesh participated in our study, which covered only nursing mothers who were at the mature milk secretion stage. Women having pregnancy with any kind of acute or chronic diseases were excluded from the study. Healthy mothers and their breastfed children (n = 55 infants age ≤ 12 months; n = 19 toddlers age 13–30 months) were included in our data analysis. Each participant was instructed to fill out a short questionnaire on anthropometric, socio-demographic, economic, and general food habits information. Anthropometric data such as nursing mothers’ body weight, height, and baby’s weight were measured at the data collection sites following standard procedure. The study was approved by the Internal Ethics Review Board at the Department of Biochemistry and Molecular Biology, School of Life Sciences, Shahjalal University of Science and Technology, Sylhet, Bangladesh (Reference number: 01/BMB/2020). A written consent form was obtained from each participant before their inclusion in the study. All the laboratory procedures were carried out following standard rules and regulations.

### Food consumption data

Nursing mothers were asked to complete a short food frequency questionnaire (FFQ) on their normal food habits. This FFQ listed typical food items consumed by the Bangladeshi population, such as rice, wheat and maize as well as groundnut, milk and milk-based products. Of these food items, only rice was regularly consumed up to three times/day by a major part of the participants. Frequency of food consumption was graded 1–5 (see Table 4, footnote).

### Sample collection and preparation

About 10–15 ml samples of breast milk and urine were collected into a sterile tube from each participant. For breast milk, we offered breast pumps to mothers, but a majority of them preferred to provide manually expressed milk. The collected urine was spot urine other than the first-morning void urine. Samples were then transported in an ice box to the laboratory and stored at -20 °C until OTA analysis. Urine creatinine content was measured by a colorimetric method according to the manufacturer's protocol (HUMAN Gesellschaft für Biochemica und Diagnostica mbH, Germany) with a semi-automatic biochemistry analyzer (Humalyzer 3000, Medicon Services, Germany). OTA concentration in milk and urine samples was determined by competitive enzyme-linked immunosorbent assay (ELISA) with a commercially available Ochratoxin A Universal ELISA kit (Helica Biosystems, Inc., Santa Ana, CA, USA, catalog no. 961OCH01LM-96). Milk and urine samples were prepared before OTA analysis according to the manufacturer’s protocol provided with the kit. Briefly, human milk samples were thawed at room temperature for 2 h before analysis. Then 250 µL of milk was added to 750 µL of absolute methanol (1:4 final dilution), mixed vigorously and allowed to stand for five minutes at ambient temperature. The samples were centrifuged at 2500 g for 10 min at room temperature (25 °C), and the supernatant was used for testing. Urine samples were thawed and centrifuged at 3030 g for 4 min at room temperature. The urine samples were then diluted with absolute methanol at 1:4 (v/v) prior to OTA analysis.

### OTA analyses in milk and urine samples

According to the manufacturer’s instructions, 200 µL of the assay diluent was dispensed into each mixing well and 100 µL of each standard or sample was added into it to prepare a diluted solution. Then, 100 µL of contents from each mixing well were transferred to the corresponding antibody-coated microtiter wells in duplicates and incubated at room temperature for 30 min. At the end of the first incubation, the contents of the wells were discarded, and the wells were washed three times with PBS-Tween wash buffer. After the washing steps, 100 µL of conjugate was added to the wells and incubated at room temperature for 30 min. After the second incubation, wells were washed and 100 µL of substrate reagent was added to each well and again incubated for 10 min. Following that, 100 µL of stop solution was added to each well and gently mixed. When the color changed from blue to yellow, the absorbance of each microwell was measured at 450 nm in an ELISA plate reader (Apollo 11 LB 913, Berthold, Germany) within 15 min. Afterwards, a calibration curve was drawn (using 0.0, 0.05, 0.1, 0.2, 0.4, and 0.8 ng/mL of OTA standard solutions) and used in order to calculate the OTA concentration based on the samples’ absorbance value. The mean of the duplicates was considered the final result. To test the accuracy of the OTA measurements, recovery studies were executed by spiking human milk or urine with two different standard concentrations of OTA: The spiked samples concentration was 60 ng/L, 100 ng/L for milk, and 50 ng/L, 100 ng/L for urine, respectively. The repeatability at those spike levels showed acceptable precisions for OTA measurements. The mean recoveries for spiked milk samples were 101.0%, 94.1%, and for spiked urines were 87.2%, and 106.6%, respectively. The limit of detection (LOD) of this ELISA method was 60 ng/L for milk and 30 ng/L for urine.

### OTA exposure of breastfed infants and risk assessment approach

The estimated daily intake (EDI) of OTA for breastfeeding babies was calculated as follows:

EDI_OTA_ (ng/kg body weight/day) = (V_BM_ × C_OTA_) / BW.

Where V_BM_: typical volume of milk consumed by an infant per day (L); C_OTA_: Concentration of OTA (ng/L); BW: body weight of the nursing babies (kg). The estimated daily consumption of milk was considered to be 150 mL per day for infants up to 2 months. For 2–4 months and older an average (50th percentile) milk intake was considered to be 185 mL per day following EFSA guidelines (EFSA Scientific Committee et al. 2017). The body weight corresponds to the individual weights of the infants in our cohort.

EDI values were then compared to a tolerable daily intake (TDI) of 17 ng/kg bw (EFSA 2006), a value close to one (14 ng/kg bw) derived by JECFA (FAO/WHO 2007) for adults. For risk assessment, the hazard quotient (HI—Hazard Index, also known as percentage of tolerable daily intake—%TDI) was calculated according to the following formula: HI = EDI/TDI.

### Statistical analysis

All data (demographic and measured OTA values) were analyzed using the software IBM SPSS Statistics version 26.0. Data were presented as percentages (%), mean, median and percentiles. Differences in the analyte concentrations between regions were analyzed by one-way ANOVA. Pearson’s correlation coefficient test (two-tailed) was applied to evaluate the correlation between milk OTA and urinary OTA level. Independent t–test was applied to assess the differences for estimated daily intake of OTA between infant and toddler groups. Spearman correlation coefficient (two-tailed) test was used to assess the correlation of OTA concentrations in breast milk and urine samples with food consumption frequencies. A level of *p*-value < 0.05 was considered statistically significant.

## Results.

### Baseline characteristics of the study subjects

The baseline characteristics of the study participants are presented in Table 1. Their average age and BMI was 25.4 ± 5.4 years and 21.8 ± 4.2 kg/m^2^, respectively. A small percentage of nursing mothers had completed their higher-secondary (5.4%) and graduation (9.5%) degrees; most of them had elementary (39.2%) and secondary (40.5%) education levels. The majority were housewives (91.9%), while 2.7% were students and the remaining subjects were private or government job holders. Regarding household status, most of the participants were in the low (47.2%) or medium (43.1%) socioeconomic group. As an aside, 68.7% had poor knowledge of diet and nutrition. The age range of infants and toddlers was 15 days-12 months and 13–30 months, respectively. Their body weight range was 2.5–10 kg (infants) and 7–16 kg (toddlers). The mean urinary creatinine level of the nursing mothers was 0.75 ± 0.53 mg/ml.

**Table 1:** Baseline characteristics of the participants

Variables	Values
Subjects (n)	74
Age (years)	25.4 ± 5.4 (18–45)
BMI (kg/m^2^)	21.8 ± 4.2 (13.8–34.2)
Creatinine (mg/ml.)	0.75 ± 0.53
Level of education, n (%)	
Illiterate	4 (5.4)
Primary	29 (39.2)
Secondary	30 (40.5)
Higher-secondary	4 (5.4)
Graduate and above graduate	7 (9.5)
Occupation, n (%)	
Students	2 (2.7)
Housewives	68 (91.9)
Office workers	3 (4.0)
Others	1 (1.4)
Occupation of spouse, n (%)	
Farmer	6 (8.1)
Office workers	16 (21.6)
Business	21 (28.4)
Others	31 (41.9)
Socio-economic status, n (%)	
Low	34 (47.2)
Medium	31 (43.1)
Upper medium	7 (9.7)
Knowledge of balanced diet & nutrition, n (%)	
Low	46 (68.7)
Medium	17 (25.4)
High	4 (6)
Children age	
Infants (15 days-12 months)	6.0 ± 3.7 (0.5–12)
15 days – 30 days	0.9 ± 0.2 (0.5–1)
31 days—2 months	1.8 ± 0.3 (1.5–2)
> 2 months—< 4 months	2.9 ± 0.3 (2.5–3.5)
4 months – 12 months	7.9 ± 2.8 (4–12)
Toddlers (13 months-30 months)	19 ± 5.0* (13–30)
Children weight (kg)	
Infants (15 days-12 months)	6.6 ± 1.8 (2.5–10)
15 days – 30 days	3.7 ± 0.9 (2.5–4.6)
31 days – 2 months	4.3 ± 1.0 (3–6)
> 2 months—< 4 months	5.7 ± 0.8 (4.8–6.8)
4 months – 12 months	7.5 ± 1.3 (5.3–10)
Toddlers (13 months-30 months)	9.3 ± 1.9* (7–16)

Values are presented as mean ± standard deviation (minimum–maximum) or n (%)

^*^ p < 0.001 when compared to infant cohort. *p* values are obtained from independent sample *t* test

in comparison between two groups

### Occurrence and levels of OTA in milk and urine samples

**Fig. 1:**
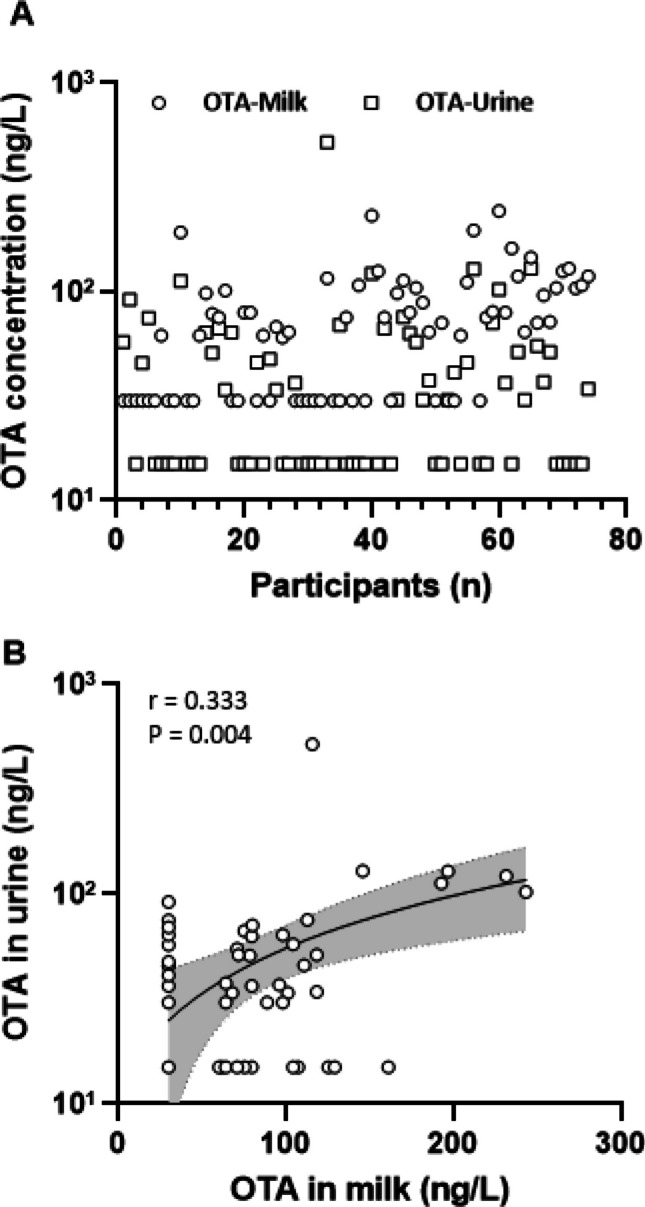
Distribution of OTA in milk and urine samples (**A**) and correlation between urinary and milk OTA levels (**B**). Logarithmic scale has been applied in Y-axis. Samples that contained OTA level below the LOD were assigned a half of LOD in depicting distribution (A) and correlation analysis (B). When considering only positive samples (≥ LOD) the co-occurrence

The detection frequency, distribution and levels of OTA in milk and urine samples of nursing mothers are shown in Table 2 and Fig. 1. OTA was detected in breast milk at a mean level of 74.8 ± 49.0 ng/L (range < LOD – 243.3 ng/L). Considering only data for samples ≥ LOD (62.2%), the mean OTA level was 102.1 ± 43.5 ng/L (range 60 – 243.3 ng/mL).

**Table 2:** Occurrence and levels of OTA in breast milk and urine samples of nursing mothers

Sample type	*n*	Positive samples *n* (% ≥ LOD)	Mean ± SD ng/L	Median ng/L	Range ng/L
Breast milk					
All regions	74	46 (62.2)	74.8 ± 49.0	69.4	< LOD – 243.3
Urine					
All regions	74	38 (51.4)	44.3 ± 63.5 (97.2 ± 142.6)^a^	30.4	< LOD – 519.3

Positive samples refer to milk or urine containing the analyte ≥ limit of detection (LOD: 60 ng/L for milk and 30 ng/L for urine)

Samples that contained OTA level < LOD were assigned a half of LOD during calculation of mean and median values

^a^creatinine adjusted OTA mean level (ng/g creatinine)

OTA was also present in urines at a mean level of 44.3 ± 63.5 ng/L (range < LOD—519.3 ng/L), and the crea-adjusted mean OTA urine level of all mothers was 97.2 ± 142.6 ng/g creatinine. Using only data for urine samples ≥ LOD (51.4%), the mean OTA level was 72.1 ± 79.5 ng/L (range 30–519.3 ng/mL). The mean OTA levels in both milk and urine samples collected in three regions were not significantly different (data not shown). The co-occurrence of OTA in milk and urine samples was 36.5%. A positive and significant correlation (*r* = 0.333, *p* = 0.004) was observed for OTA in milk and urine samples (Fig. 1).

### OTA intake estimates

The average estimated daily intake (EDI) for OTA in all infants was 15.0 ± 8.3 ng/kg bw with a wide range between 4.5 and 45.0 ng/kg bw (Table 3). The mean OTA intake was slightly higher in 1–2 months old infants (16.2 ± 7.8 ng/kg bw/day) than in the other infant age groups or the toddlers (12.6 ± 8.1 ng/kg bw/day), yet differences in EDI were not statistically significant. Of note, the EDI calculated here for toddlers is not a reliable estimation, since this age group (> 13 months) suckles breast milk only occasionally whilst ingesting mainly post-weaning food, which may contain higher levels of OTA than human milk.

**Table 3:** Estimated daily intake of OTA (ng/kg b.w./day) for nursed infants and toddlers

Group	N	Mean ± SD (ng/kg)	Median (ng/kg)	Range (ng/kg)	95% Cl for mean		Exceeding PTDI^#^, n (%)	HI (% TDI)				
					Lower bound	Upper bound		Mean	Median	Max	95% Cl for mean	
											Lower bound	Upper bound
Infants (all)	55	15.0 ± 8.3	13.3	4.5–45.0	12.8	17.3	19 (34.5)	0.88 (88)	0.78 (78)	2.65 (265)	0.75 (75)	1.02 (102)
15 days – 30 days	5	12.8 ± 5.2	15.6	6.5–17.8	6.3	19.2	1 (20)					
31 days – 2 months	6	16.2 ± 7.8	14.9	7.8–29.8	8.0	24.5	3 (50)					
> 2 months – < 4 months	7	12.6 ± 5.8	13.1	4.5–18.7	7.8	17.8	2 (28.6)					
4 months – 12 months	37	15.6 ± 9.1	13.3	5.5–45.0	12.6	18.6	13 (35.1)					
Toddlers (all)	19	12.6 ± 8.1	11.4	5.5–42.8	8.9	16.6	2 (10.5)	0.78 (78)	0.67 (67)	2.52 (252)	0.52 (52)	0.98 (98)

Infants: age ≤ 12 months; Toddlers: age 13–30 months. Milk samples that contained OTA level below the LOD (60 ng/L) were assigned a half of LOD during EDI calculation

^#^Provisional tolerable daily intake (PTDI) = 17 ng/kg b.w./day (or 120 ng/kg b.w./week; (European Food Safety Authority 2006)

When comparing EDI values for OTA in the infant group with the provisional TDI value set by EFSA (EFSA 2006), 19 of 55 infants (34.5%) exceeded this PTDI of 17 ng/kg bw. Furthermore, one may consider to apply a lower TDI of one-third for infants below 4 months of age, in line with the ‘Guidance on the risk assessment of substances present in foods intended for infants below 16 weeks of age’ (EFSA Scientific Committee et al. 2017). The calculated mean HI for OTA was 0.88 (median 0.78 and max 2.65) in infants and 0.78 (median, 0.67 and max 2.52) in toddlers. The 95% Cl of lower and upper bound were 0.75 and 1.02, respectively, in infants and 0.52 and 0.98, respectively, in the toddler group. Further aspects related to risks from OTA exposure in infants with breast milk are addressed in the Discussion.

### Correlation between maternal OTA biomarker levels and food consumption

Possible associations between the individual maternal food consumption pattern and OTA levels found in milk and in urine samples were investigated with regard to major food items such as rice, wheat/maize, milk and milk products and groundnut in Spearman correlation analysis (Table 4). No significant correlations were found between OTA levels in breast milk samples and consumption frequency for rice (*p* = 0.579), wheat/maize (*p* = 0.884), milk and milk-based products (*p* = 0.062) or groundnut (*p* = 0.056). Similarly, no significant correlation was observed between urinary OTA levels and consumption of any of the listed food items.

**Table 4:** Correlation of OTA concentrations and maternal food consumption frequency^#^

Food items	OTA in milk		OTA in urine	
	Correlation (*r*)	*p*-value	Correlation (*r*)	*p*-value
Rice	-0.084	0.579	0.273	0.098
Wheat/maize	-0.022	0.884	-0.009	0.957
Milk and milk products	0.278	0.062	-0.072	0.668
Groundnut	0.2502	0.056	0.022	0.895

^#^Assessment of food consumption frequency was done using numerical scores for the following food items;

for rice: 1 = 1 time/day, 2 = 2 times/day, 3 = 3 times/day; wheat/maize: 1 = 1 time/day, 2 = 2–3 times/week,

3 = do not consume at all, 4 = weekly; milk and milk based products: 1 = every day, 2 = 2–3 days per week,

3 = monthly, 4 = weekly, 5 = do not consume at all; groundnut: 1 = 1 time/day, 2 = monthly, 3 = not at all; 4 = weekly

Positive samples (≥ LOD) were considered for correlation analysis

*P-*values are obtained from Spearman’s correlation coefficient (two-tailed)

## Discussion

In Bangladesh, since toxic contaminants are not routinely monitored in foods, there are only limited data on the presence of mycotoxins in food commodities (Islam and Hoque 2013). This hampers a conventional exposure assessment, but biomarker-based studies can provide useful information on exposure to mycotoxins in different groups of the Bangladeshi population. A recent study reported aflatoxin M_1_ (AFM_1_) presence in > 50% of human breast milk samples in Bangladesh (Islam et al. 2021) and corroborates earlier biomarker findings on AFM_1_ in urines (Ali et al. 2017b; Gerding et al. 2015). Furthermore, OTA has been detected frequently in urines of adults, children, and pregnant women in Bangladesh (Ali et al. 2016a, b; Ali and Degen 2020; Kyei et al. 2022). However, there is no data as yet on OTA exposure of nursing mothers and their children. This is the first study that determined OTA both in human milk and maternal urine samples collected in three different districts in Bangladesh.

In this cross-sectional study with 74 participants, OTA was detected in 62.2% of their breast milk (range < LOD to 243.3 ng/L), and in 51.4% of their urines (range < LOD to 519.3 ng/L). The frequent detection of OTA in both sample types (Table 2) clearly indicates a widespread dietary exposure to mycotoxin in our cohort of nursing mothers. Some regional variation observed in detection frequency and levels of OTA biomarkers may reflect differences in dietary habits and/or mycotoxin contamination of consumed foods. Moreover, in individuals with quite stable food habits, the OTA biomarker levels in breast milk or urine are known to fluctuate over time (Muñoz et al. 2014; Ali et al. 2016a). Despite such inherent uncertainties, the urinary OTA levels in nursing mothers (mean 0.04 ng/mL, max 0.52 ng/mL) can be related to other studies conducted in Bangladesh in a pregnant women cohort in Dhaka (mean 0.10 ng/ml, max 0.84 ng/ml; Ali et al. 2016b) and Sylhet region (mean 0.32 ng/ml, max 2.93 ng/ml; Kyei et al. 2022), children cohorts in Rajshahi and Dhaka region (mean 0.11 ng/ml, max 0.75 ng/ml; Ali and Degen 2020) and an adult cohort in Rajshahi region (mean 0.21 ng/ml, max 6.10 ng/ml; Ali et al. 2016a). Overall, the OTA results in urines document widespread dietary mycotoxin of the Bangladeshi population. Higher biomarker levels found in several of the above studies can be related to urine sample preparation with enzymatic hydrolysis of OTA conjugates that occur along with OTA aglycone (Muñoz et al. 2017), whilst the present analysis by ELISA detects only the latter. Either way, both approaches need to consider that only a rather small fraction (< 3%) of the ingested OTA is excreted with urine (Degen 2016).

Several biomonitoring studies reviewed elsewhere (Duarte et al. 2011; Ropejko and Twarużek 2019) have determined OTA in human blood as it has a long half-life (about 35 days) due to its high affinity for serum proteins. But, blood sampling is an invasive procedure and less often applied in field studies, although OTA in plasma or serum is a good long-term biomarker of exposure to this mycotoxin. Some OTA in the circulating blood of nursing women is continuously transferred to breast milk. The lactational transfer was investigated in a longitudinal study that determined OTA milk/plasma (M/P) ratios at various stages of breastfeeding: In the first days after parturition, a high fraction (about 40%) of OTA in maternal plasma occurred in colostrum, whilst at later stages OTA concentrations were about one quarter (M/P ratio 0.25) of those measured in plasma (Muñoz et al. 2014). In other words, when the mother ingests contaminated food, this is also reflected in OTA occurrence in human milk.

OTA is a food and feed contaminant with global occurrence, albeit the levels in food commodities in various parts of the world are known to vary considerably. Therefore, it is no surprise that a very wide range is also observed for OTA concentrations measured in human milk samples from different countries. An overview in Table 5 on results and applied analytical methods allows to view our first data set on OTA levels in Bangladesh in a larger context of studies conducted in Africa, Asia, Europe, and South America. Rather than discussing all data in detail, some more general aspects are of interest: OTA concentrations in human milk samples (mean levels, ranges) from African countries are among the highest values reported, for example, in Egypt and Nigeria (Table 5). As elaborated in the respective publications or recently by Duarte et al. (Duarte et al. 2022), such breast milk levels will result in OTA exposures of nursed infants that are clearly of concern. Furthermore, when data of multiple studies are available, as for Egypt, the outcome can vary. This is likely due to the collection of milk samples in different years and/or in cohorts from various parts of the country. Likewise, studies conducted on the Asian continent also show differences in OTA breast milk levels: two studies in Iran found low ranges (< 5–16.4 ng/L, (Afshar et al. 2013) and 1.6–60 ng/L, (Dehghan et al. 2014) whilst high values (mean 1990 ng/L, range 110–7340 ng/L) were reported in a more recent study (Kamali et al. 2017). The picture varies also in Turkey, with low (25 ng/L, Altun et al. 2019), moderate (140 ng/L, Uyar et al. 2014) and high (> 1500 ng/L, Gürbay et al. 2010) mean OTA levels in three studies. Differences in OTA milk levels and its prevalence point to differences in dietary mycotoxin exposure of nursing women that is related to their dietary habits, region of residence, sampling time (year) and contamination of locally produced foods; the latter depending on the climate in a given geographical location (Cherkani-Hassani et al. 2016; Warth et al. 2016; Coppa et al. 2019).

**Table 5:** Worldwide occurrence and concentrations of OTA in human breast milk samples (from 1988—2023)

Country	Milk type	Dietary Inform	Frequency (%)	Mean ± SD (ng/L)	Concentration range (ng/L)	Analytical method, sample preparation	LOD/LOQ (ng/L)	Reference
**Africa**								
Angola	Mature	FQ	37/37 (100)	700 ± 475	181–2527	ELISA	150/-	(Duarte et al. 2022)
Egypt	NI	NI	3/10 (30)	8870	3000–15000	HPLC	-	(El-Sayed et al. 2000)
Egypt	NI	NI	43/120 (35.8)	21,100 ± 13,700	5070–45010	HPLC	100/-	(El-Sayed et al. 2002)
Egypt	Mature	NI	36/50 (72)	1890 ± 980	NI	HPLC	NI	(Hassan et al. 2006)
Morocco	Colostrum	FQ	45/82 (55)	2170 ± 2790	80–10040	ELISA, SkM	80 /-	(Cherkani-Hassani et al. 2020)
Nigeria	Mature	FQ	49/75 (65)	9500 ± 16,000	800–68000	LC–MS/MS	800/-	(Braun et al. 2022)
**Asia**								
Bangladesh	Mature	FQ	46/74 (62.2)	74.8 ± 49.0	< LOD-243.3	ELISA, SkM	60/-	Present study
India (northern)	Mature	FQ	13/100 (13)	-	7.8–39.8	ELISA	15.6/31.3	(Mehta et al. 2021)
Iran	Mature	FQ	5/136 (3.7) 2/136 (2.72)	-	< 5–16.4	ELISA, SkM HPLC-FD, LLE	NI	(Afshar et al. 2013)
Iran	NI	NI	84/87 (96.6)	25 ± 14	1.6–60	ELISA, SkM	NI	(Dehghan et al. 2014)
Iran		NI	84/84 (100)	1990 ± 1340	110–7340	ELISA	62/-	(Kamali et al. 2017)
Turkey	NI	NI	75/75 (100)	> 1500	621–13,110	LC-FD, LLE	10/NI	(Gürbay et al. 2010)
Turkey	Mature	NI	34/70 (48.6)	140 ± 30	-	ELISA	-	(Uyar et al. 2014)
Turkey	All	NI	21/92 (22.8)	25.4 ± 4.1	10–100	ELISA	0.01/NI	(Altun et al. 2019)
**Europe**								
Austria	All	NI	12/15 (80)	3.0	< LOQ-4.6	LC–MS/MS	-/1.5	(Braun et al. 2020)
Germany: NRW LS	All	NI	21/30 (70) 25/60 (42)	24 ± 21 14 ± 15	< LOD-100	HPLC–MS	10/30	(Muñoz et al. 2013)
Germany	NI	NI	4/36 (11)	-	17–30	HPLC	10/-	(Gareis et al. 1988)
Hungary	Colostrum		38/92 (41)	-	200–7630	HPLC	NI	(Kovács et al. 1995)
Italy	All	FQ	22/111 (20)	-	100–12000	HPLC	100/-	(Micco et al. 1995)
Italy	Colostrum	FQ	198/231 (86)	6 ± 8	1–57	HPLC	0.5	(Turconi et al. 2004)
Italy	Mature	FQ	61/82 (74)	30 ± 67	< 5–410	HPLC, IAC	2/5	(Galvano et al. 2008)
Italy	Colostrum	FQ	45/57 (78.9)	10 ± 16	1–75	HPLC	0.5/1	(Biasucci et al. 2011)
Norway	NI	NI	38/115 (33)	NI	10–130	HPLC	10	(Skaug et al. 1999)
Norway	Mature	FQ	17/80 (21)	30	10–182	HPLC	10	(Skaug et al. 2001)
Poland	All	NI	40/78 (51)	19 ± 8	22–26.4	ELISA	NI	(Karwowska et al. 2004)
Poland	Colostrum	NI	5/13 (38)	6 ± 4	6–17	HPLC	5/15	(MatkI et al. 2006)
Slovakia	Mature	NI	23/76 (30)	-	LOQ-60	HPLC	5/14	(Dostal et al. 2008)
Sweden	Mature	-	23/40 (58)	-	10–40	HPLC	10/40	(Breitholtz-Emanuelsson et al. 1993)
**South America**								
Bolivia	Mature	NI	13/29 (44.8)	53 ± 33	20–146	HPLC, IAC	18/36	(Ferrufino-Guardia et al. 2019)
Brazil	Transitional	NI	4/74 (5)	360	18–2600	UPLC-MS/MS	50/170	(Coppa et al. 2021)
Brazil	NI	NI	66/100 (66)	-	0.8–21	LC-FD, IAC	0.3/0.8	(Iha et al. 2014)
Brazil	NI	FQ	2/50 (4)	-	11–24	HPLC-FD, IAC	-/10	(Navas et al. 2005)
Chile	Colostrum	NI	9/9 (100)	110 ± 45	40–184	LC-FD, LLE	10/30	(Muñoz et al. 2010)

*ELISA *Enzyme-linked immunosorbent assay, *FQ* Food questionnaire, *HPLC-FD* High performance liquid chromatography with fluorescence detector, *IAC* Immuno-affinity cartridge, *LC-FD *Liquid chromatography with fluorescence detector, *LC–MS/MS *Liquid chromatography-tandem mass spectrometry, *LLE* Liquid − liquid extraction, *LOD* Limit of detection, *LOQ* Limit of quantification, *LS* Lower Saxonia, *NRW* North-Rhine Westphalia, *NI* No information provided, *SkM* Skimmed milk, *UPLC-MS/MS* Ultra performance liquid chromatography-tandem mass spectrometry

OTA levels in breast milk from Bangladesh (range < LOD–243 ng/L, mean 74.8 ng/L) are higher than those reported in a study from northern India (range 8–40 ng/L, (Mehta et al. 2021) where rice is also a staple food. Yet, in both countries, these are singular studies, and follow-up is indicated due to the many variables which impact on dietary OTA exposure. Studies conducted between 1998 and 2020 in several European countries observed lower OTA breast milk levels than in the present study, with the exception an early study in Italy (Micco et al. 1995) and one in Hungary on colostrum (Kovács et al. 1995). The nowadays rather good situation in Europe is in part related to rather strict surveillance of mycotoxin contaminants in foods based on legislation setting maximum permissible levels in various commodities (Commission Regulation 2023). Similar regulations for mycotoxins exist in other countries, including foods intended for infant consumption (Coppa et al. 2019). Yet, enforcement by regular analytical controls is often lacking which is the case in Bangladesh (Islam and Hoque 2013) and in many other developing countries. Now some remarks on studies from South America: OTA analysis of samples from milk banks in Brazil found low levels (Navas et al. 2005; Iha et al. 2014), whilst a recent study detected higher levels, but only in 4 of 74 milk samples from women recruited in a municipal health care center (Coppa et al. 2021). In a longitudinally designed study in Chile, OTA levels varied between breastfeeding periods, with the highest levels found in colostrum (Muñoz et al. 2010, 2014). Overall, data reported for OTA concentrations in human milk (Table 5, and Fig. 3 in (Cherkani-Hassani et al. 2016) document that, as a consequence of high or low maternal mycotoxin exposure, also OTA intake of nursed infants worldwide varies considerably.

Regarding sources of OTA intake in Bangladeshi nursing women, one should consider rice, a main staple food of the Asian population, including Bangladesh; rice can be contaminated by OTA and the related nephrotoxic mycotoxin citrinin (Ali 2018). A few surveys in Bangladesh also report OTA presence in maize, cereals, and in animal feed which could give rise to OTA in animal milk (Al-Mamun et al. 2002; Dawlatana et al. 2002, 2008). In our study, an attempt was made to correlate the OTA biomarker levels in lactating mothers with their consumption frequency of major food items. No significant association was found for breast milk and urinary OTA with maternal food consumption (Table 4). However, we could not collect information on spices and other food items, which may also contribute to the total mycotoxin exposure of our study participants.

Regarding exposure of nursed infants in Bangladesh, OTA concentrations determined in breast milk samples served to estimate the daily intake (EDI) of babies in several age groups (Table 3) using average volumes of breast milk consumption and individual body weights in the calculation (see Methods). The OTA intake of all infants ranged between 4.5 and 45 ng/kg b.w./day, and their mean exposure (EDI of 15 ng/kg b.w.) was slightly higher than in the toddler group (mean 12.6 ng/kg b.w.). Yet, as noted above, we consider the EDI for toddlers not as reliable since they suckle breast milk occasionally but ingest mainly post-weaning food. To provide a perspective on the risks of OTA exposure for nursed infants, we compared EDI values to provisional tolerable daily intake (PTDI) values of 17 ng/kg b.w. Considering these PTDI values, then 34.5% of the infants (up to 12 months) and 10.5% in the toddler group (> 13 months) exceed this limit. The findings can be also expressed by the Hazard Index (HI = EDI/TDI. When the HI is < 1, the exposure is below; when > 1, it exceeds the chosen TDI. In our study the calculated mean HI for OTA was 0.88 (median 0.78 and max 2.65) in infants and 0.78 (median 0.67 and max 2.52) in toddlers. The lower and upper bound of HI were 0.75 and 1.02, respectively, in infants and 0.52 and 0.98, respectively, in the toddler group. Either way, the assessment indicates that part of the nursed infants in our study are exposed to undesirable levels of OTA. On the other hand, breast milk sample analysis in Bangladesh found concentrations in the range of < LOD to 243 ng/L (Table 2), which are well below the maximum permitted level for OTA of 500 ng per kg or L in commercial baby foods (Commission Regulation 2023). Occurrence of mycotoxins, including OTA, in infant formula and other products has been recently reviewed, and the data show in some samples an exceedance of the regulatory limits for mycotoxins (Coppa et al. 2019; Hernández et al. 2021). Although the high EDI values calculated for OTA in breastfed infants in some countries may be lowered by providing infant formula, this is usually not a viable alternative in developing countries with limited financial resources (Hernández et al. 2021). Considering the well-known benefits of human milk, breastfeeding should not be discouraged; instead, steps should be taken to improve food safety and thereby minimize human exposure to mycotoxins (Muñoz et al. 2014).

The present study has some limitations. First, though our study covers three regions of Bangladesh, the number of participants (n = 74) was relatively small. Therefore, the study findings might not reflect the entire scenario of Bangladesh. Second, we collected milk and urines samples only once, which precluded to investigate of possible seasonal effects on the OTA biomarker levels among the participants. Third, we applied ELISA for OTA determination in biofluids, which is well suited for breast milk analysis but may have underestimated total OTA occurrence in urine (including glucuronides), as it detects only unconjugated OTA. Applying sensitive LC–MS/MS multi-biomarker methods could help to further explore also concurrent exposure to other mycotoxins in milk and urines of nursing mothers in Bangladesh. However, our present findings on OTA provide a basis for follow-up studies in this particular population group.

In summary: This study provides evidence for OTA exposure in Bangladeshi lactating mothers: OTA was detected in almost two-thirds of breast milk and half of urine samples. Although the mean level of OTA measured in breast milk was considered moderate, an estimated daily exposure for several infants exceeded limits for a tolerable intake of this nephrotoxic mycotoxin. As the analysis in the present study is limited to a small number of samples, additional biomonitoring studies with larger sample size are recommended to gain further insight on breastfeeding mothers and children exposure to OTA in Bangladesh. Moreover, regular surveillance of dietary contaminants is required to identify major sources of OTA intake in the Bangladeshi population.

## Data Availability

All relevant data are included in the article.
